# Mapping current research and identifying hotspots of ferroptosis in cardiovascular diseases

**DOI:** 10.3389/fcvm.2022.1046377

**Published:** 2022-11-04

**Authors:** Teng Teng, Chun-Yan Kong, Rong Huang, Zhen-Guo Ma, Can Hu, Xin Zhang, Min Hu, Qi-Zhu Tang

**Affiliations:** ^1^Department of Cardiology, Renmin Hospital of Wuhan University, Wuhan, China; ^2^Hubei Key Laboratory of Metabolic and Chronic Diseases, Wuhan, China

**Keywords:** ferroptosis, cardiovascular diseases, bibliometric analysis, heart, ischemic-reperfusion injury

## Abstract

**Objective:**

Ferroptosis is a unique cell death depended on iron metabolism disorder which is different from previous apoptosis-regulated cell death. Early studies have proposed that ferroptosis is closely associated with multiple cardiovascular diseases (CVDs). However, the relationship of ferroptosis and CVDs has not been summarized by using bibliometric analysis. We intended to illustrate the development of ferroptosis in CVDs over the past years and provide relevant valuable information.

**Materials and methods:**

The authoritative database of Web of Science Core Collection was collected for retrieving ferroptosis studies in CVDs. In this work, statistical and visualization analysis were conducted using VOSviewer and Citespace.

**Results:**

A total of 263 studies were included in the final study. From the perspective of the overall literature, the study maintains an increased trend year by year and most manuscripts belonged to original article. China was the most productive country with the utmost scientific research output, as well as the institutions and authors, followed by Germany and the United States of America (USA). Jun Peng from China contributes to the most publications. Collaborative efforts between institutes and authors were limited and there was little widespread cooperation. In addition, burst keywords analysis discovered that ischemia-reperfusion (I/R) injury, heart failure (HF), and atherosclerosis were the top three research directions of ferroptosis in CVDs. The burst investigation and timeline views also indicated that endothelial injury and gut microbiota may also serve as new research topics in the future.

**Conclusion:**

This study provided comprehensive and specific information about the most influential articles on ferroptosis in CVDs. The relationship between ferroptosis and CVDs had attracted the scholar’s concerns especially in China. Cooperations and communications between countries and institutions should be emphasized and future directions can be concentrated on endothelial disorder and gut microbiota.

## Introduction

Ferroptosis belongs to a new kind of cell death characterized by iron ion-related metabolic disorder. The incidence of ferroptosis and subsequent series reactions can lead to a reduced antioxidant capacity, excessive lipid reactive oxygen species (ROS) accumulation, and eventually oxidative cell death (1). The process of ferroptosis was regulated by specific genes and molecules which were different from previous common cell death, such as apoptosis, necrosis, pyroptosis, and autophagy (2). The disorder of ferroptosis such as iron metabolism disorder, oxidative stress, descending glutamate synthesis, and lipid peroxidation had provided a new target for the intervention of iron ferroptosis-associated diseases. Previous studies had demonstrated that ferroptosis was strongly associated with the development of many diseases ranging from tumor growth, nervous system degeneration, blood system, endocrine, and metabolic system (3). At the same time, numerous studies of abnormal cell death had proved that ferroptosis was closely associated with the incidence of CVDs and the dynamic equilibrium of iron metabolism was essential for normal cardiac function maintenance (4). Too much iron deposition in the cytoplasm can directly result in vasodilation dysfunction, deterioration of atherosclerotic plaques, and the incidence of arrhythmias, and even heart failure (HF) (5).

Early studies had revealed that elevated iron in the myocardium can lead to seizure-connected cardiac ferroptosis, also called as “Epileptic Heart,” which was considered as the most serious threat to sudden unexpected death during epilepsy (SUDEP) (6). Additionally, excess iron elevated neuro-cardiogenic sensibility and occurrence of abnormal cardiac electrical abnormality accompanied with sympathetic hyperactivity (7). In addition, iron disorder can also reflect the extent of left ventricular remodeling after myocardial infarction. The overaccumulation of iron around the area of infarction with elevated T2 values was associated with adverse LV remodeling and incidence of intramyocardial hemorrhage (IMH) on cardiac magnetic resonance (8). Cellular acidosis induced by changes in the internal environment was thought to trigger the too much production of ferrivalent or ferrous ions and subsequently resulted in the iron-associated Fenton reaction, ROS production, oxidative stress response and ferroptosis in cardiomyocytes (9, 10). Consequently, further investigation of the mechanisms regulating iron metabolism and ferroptosis in CVDs might promote both diagnosis and treatment in CVDs.

Currently, most research and articles are based on manual searching and personal experience, lacking a thorough overview and a holistic approach. Bibliometric analysis can evaluate the scientific development and trend based on special algorithms (11). Bibliometric study can be used to provide a clear presentation of countries/regions, authors, institutions, and keywords during the development in a certain field. The result will also explore the current status, hot topics, and dynamic trends. Bibliometric views have reported a correlation between ferroptosis and cancer treatment and stroke (12–14), but no research has been conducted on the relationship between ferroptosis and CVDs. This study aimed to review the status and emerging trends of ferroptosis in CVDs and provide research trend for scholars in the application of ferroptosis in the future.

## Materials and methods

### Search strategies

Literatures in this study were searched from the Web of Science core collection database (WoSCC) which was regarded as the most authoritative citation with powerful indexing functions (15). The timeline was confined from the earliest time of the database to the latest time of literature search (July 25, 2022). All the types of literatures were primarily analyzed in the study with the search formula of TS = (“cardiovascular” OR “heart” OR “circulation”) AND TS = (“ferroptosis” or “Iron death”). Only literature was restricted to articles and reviews requiring independent correction by two researchers undergoing further study.

### Data collection and analysis

All the data were analyzed and visualized by using the Microsoft Excel 2019, VOSviewer and CiteSpace. Microsoft Excel (v. 2019) was used to calculate the total and annual publications and citations. The distribution and collaborative relationships of authors, countries and institutions were constructed by VOSviewer. Moreover, the keywords with strong citation and burst detection were classified into several clusters. We also use CiteSpace to conduct strong citation bursts of co-reference and keywords as well as the timeline view.

## Results

### Publication outputs and temporal trend

**FIGURE 1:**
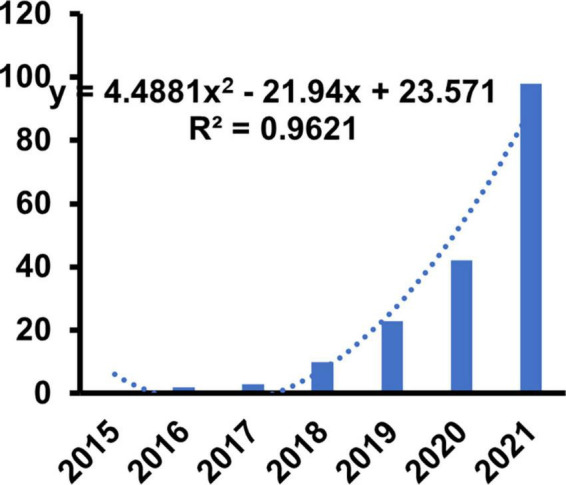
Annual publication of the ferroptosis in cardiovascular disease.

Web of science had collected 276 items on ferroptosis with CVDs with [TS = (“cardiovascular” OR “heart” OR “circulation”) AND TS = (“ferroptosis”)]. Of these, 164 (59.4%) were research articles, 99 (35.9%) were reviews, and 8 (2.9%) were early access. Detailed information of articles was presented in Table 1. Only articles and reviews were included in the further study. As many as 263 items were included in the final study. Although the research of ferroptosis was first proposed in 2012, the annual production of articles has achieved a steady growth from 1 in 2015 to 84 in 2022. We also constructed the model to predict the trend based on the output of 2015–2022. We discovered that the increased trend was best proportion to the polynomial function with the highest R^2^ (0.9621). The specific forecasting equations were *y* = 4.4881 × 2–21.94x + 23.571 implying that research in this field was on the rise and had achieved ever-increasing attention (Figure 1).

**TABLE 1:** Summary and classification of included studies.

Publication type	Counts	%
Article	164	59.4
Review	99	35.9
Early access	8	2.9
Book chapter	2	0.7
Revised/Editorial material/Meeting abstract	3	1.1

### The distribution of countries

**FIGURE 2:**
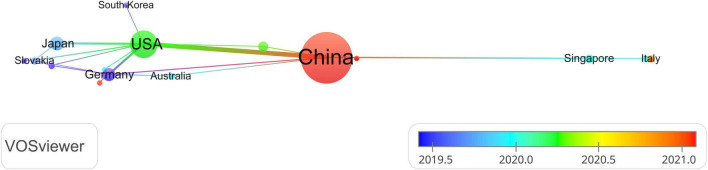
Distribution of authors.

A total of 36 countries and 400 organizations contributed documents in the area. The top 10 high-yield countries and institutions were depicted in Table 2. China (189 71.9%) was the country with the most publications followed by the USA (54 20.5%) Japan (14 5.3%), Germany (12 4.6%), and Singapore (5 1.9%). The association among countries was constructed by the VOSviewer software. The node in the visualization map was almost equal to the output of publications, while the line was equal to the extent of closeness and exchange. Figure 2 presented the network and collaborations of countries, and we can infer that China was the core in this field, followed by UAS, Germany, and Japan. Furthermore, the cross-country collaboration also demonstrated that only those dominant countries cooperated, and there was still a wide potential for communication and cooperation in this field.

**TABLE 2:** Specific information of the top 10 countries and institutions.

Rank (country)	Source	Publications	Citations	Rank (institution)	Source	Country	Publications	Citations
1	China	189	4311	1	Central South University	China	15	206
2	USA	54	3979	2	Zhejiang University	China	13	877
3	Japan	14	575	3	Fudan University	China	11	121
4	Germany	12	1158	4	Shanghai Jiao Tong University	China	10	561
5	Canada	7	197	5	Wuhan University	China	8	154
6	Singapore	5	344	6	Jinan University	China	6	82
7	Slovakia	4	144	7	Nanchang University	China	6	24
8	Italy	4	110	8	University of South China	China	6	40
9	France	3	64	9	Wenzhou Medical University	China	6	66
10	Turkey	3	29	10	Keio University	Japan	6	201

### Organizations distribution

**FIGURE 3:**
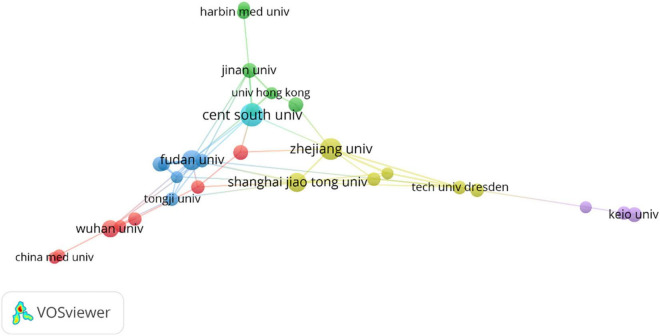
Distribution of institutions.

From the perspective of publishing organizations, Central South University ranked first in publishing articles, followed by Zhejiang University and Fudan University. Almost all the top ten organizations were from China and Table 2 provided detailed information. Based on these results, the study of ferroptosis and CVDs in these countries played a pivotal role and China still occupied a pivotal position. From the composition of publishing organizations, there were mainly four clusters in this field (Figure 3). Yellow clusters mainly include Zhejiang University, Shanghai Jiao Tong University, University of Pittsburgh, University of California, Davis and Kyoto University. The red cluster was composed of Harvard University, Harvard Medical School, University of Oxford, University of Washington, University of Tokyo, Technical University of Munich, and Imperial College London. The blue cluster mainly consists of University Medical Center Hamburg-Eppendorf, Leiden University, Universiteit van Amsterdam, Technische Universitaet Dresden, and University of Cologne. The yellow cluster mainly includes the German Center for Cardiovascular Research (DZHK), Heidelberg University, Southwest Medical University, and Gottingen University. The pink clusters mainly include Tampere University and George Washington University. Several clusters were scattered across the world, and it can be suggested that intense communication was urgently needed in this field.

### Authors distribution

**FIGURE 4:**
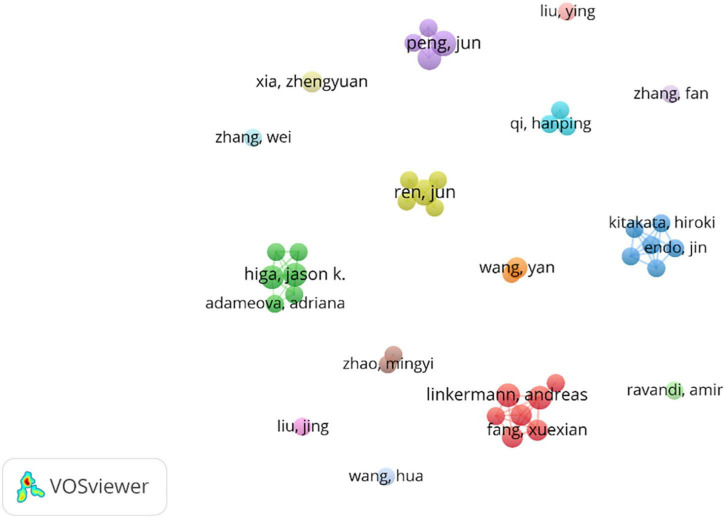
Distributions of authors.

A total of 5,486 authors published articles on ferroptosis and CVDs (Table 3). J Peng, from the Department of Pharmacology, Xiangya School of Pharmaceutical Sciences, Central South University, and J. Ren, from the Department of Cardiology, Shanghai Institute of Cardiovascular Diseases, Zhongshan Hospital, Fudan University, had published the most papers (6, 2.2%), followed by Wang H., A. Linkermann, J. K. Higa, X. J. Luo, T. Matsui (5 1.8%). Just as the number of the documents, the author from China still occupied an overwhelmingly role. As shown in Figure 4, the authors in ferroptosis and CVDs were scattered and loose, and some authors cooperated with each other, but no large-scale cooperation had yet been formed. The red cluster mainly includes X. Fang, F. Gao, and J. Li. The green cluster mainly includes A. Adameova, Y. Baba, and J. K. Higa. The blue cluster mainly includes J. Endo, Y. Katsumata, and H. Kitakata.

**TABLE 3:** Summary of the top 10 authors.

Rank	Author	Country	Documents	Citations	Average citation/Publication
1	Jun Peng	China	6	120	20
2	Jun Ren	China	6	35	5.8
3	Hao Wang	China	5	872	174.4
4	Andreas Linkermann	Germany	5	1050	210
5	Jason K. Higa	USA	5	255	51
6	Xiu-Ju Luo	China	5	113	22.6
7	Takashi Matsui	USA	5	255	51
8	Xuexian Fang	China	4	850	212.5
9	Junxia Min	China	4	850	212.5
10	Zhengyuan Xia	China	4	117	29.25

### Journals distribution

A total of 263 articles from 142 documents were retrieved in our study. There were 272 papers in the top 10 journals which almost occupied 35% in the total literature. Frontiers in Cell and Developmental Biology had the most outputs (15, 5.7%), followed by Frontiers in Pharmacology (10, 4.1%) and Frontiers in Cardiovascular Medicine (10, 3.8%). Not all the journals belong to the cardiovascular area, which implied extensive investigations was needed between ferroptosis and CVDs. Theranostics was the journal with the highest impact factor in the top 10 journals. Most journal was distributed in Q2 and Q3 region. The specific information of journals distribution was shown in Table 4.

**TABLE 4:** Top 10 journals of ferroptosis in cardiovascular disease.

Rank	Source	IF	Publications	Citations	Average citation/Publication	JCR (2021)
1	Frontiers in cell and developmental biology	6.081	15	52	3.47	Q2
2	Frontiers in pharmacology	5.988	11	170	15.45	Q2
3	Frontiers in cardiovascular medicine	5.846	10	34	3.4	Q2
4	Oxidative medicine and cellular longevity	7.310	9	67	7.44	Q2
5	Biochemical and biophysical research communications	3.322	6	209	34.83	Q3
6	Free radical biology and medicine	8.101	6	103	17.17	Q2
7	Cell death and disease	9.685	5	170	34	Q1
8	Biomedicine and pharmacotherapy	7.419	4	237	59.25	Q2
9	American journal of physiology heart and circulatory physiology	5.125	22	798	39.27	Q2
10	Theranostics	11.600	4	156	39	Q1

### Analysis of co-cited authors and journals

**FIGURE 5:**
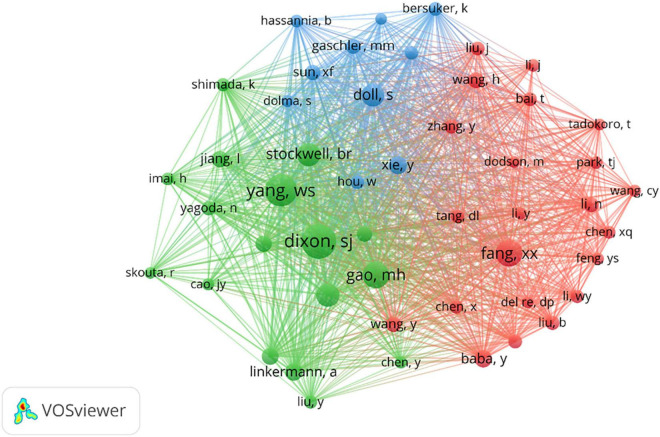
Distribution of co-cited authors.

The influence of the article was also reflected by the co-citation frequency. Among the total 12,088 authors, 7 authors had been cited over 100 times. S. J. Dixon from the Department of Biology, Stanford University, Stanford, was the top co-cited author with 248 citations followed by W. S. Yang from the Department of Biological Sciences St John’s University Queens, USA. Although China had published the most articles in CVDs-related ferroptosis, most co-cited authors were from the USA, Germany, and China. As was shown in Table 5, Cell (717) was the top cited journal, followed by Nature (570) and Proceedings of the National Academy of Sciences (548). According to the order of the co-cited documents, Nature had the highest IF (113.915), cell (IF = 66.85) and circulation (IF = 39.918) ranked second and third place. There were mainly three clusters from the co-cited authors, as can be seen in Figure 5.

**TABLE 5:** Information of top 10 co-cited authors and journals.

Rank	Author	Citations	Country	Rank	Journal	IF	Citations	JCR (2021)
1	Scott J. Dixon	248	USA	1	Cell	66.850	717	Q1
2	Wan Seok Yang	212	USA	2	Nature	69.504	570	Q1
3	Xuexian Fang	150	China	3	Proceedings of the national academy of sciences	12.779	548	Q1
4	Minghui Gao	139	USA	4	Free radical biology And medicine	8.101	531	Q2
5	José Pedro Friedmann Angeli	109	Germany	5	Journal of biological chemistry	5.486	466	Q2
6	Brent R Stockwell	108	USA	6	Cell death and differentiation	12.067	358	Q1
7	Sebastian Doll	107	Germany	7	Biochemical and biophysical research communications	3.322	356	Q3
8	Y Xie	66	China	8	Circulation research	23.213	335	Q1
9	Andreas Linkermann	63	Germany	9	Circulation	39.918	331	Q1
10	Yuichi Baba	61	USA	10	Cell death and disease	9.685	328	Q1

### Analysis of co-cited references and references burst

**FIGURE 6:**
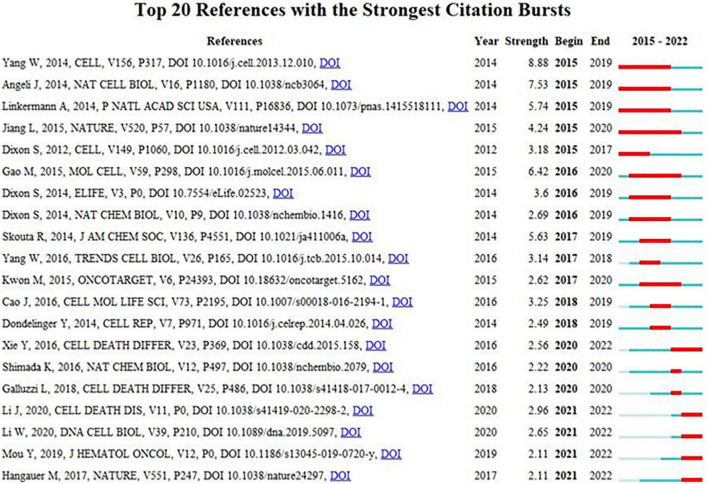
The distribution of top 20 references with the strong citation bursts.

The top 10 most frequently cited references in ferroptosis and CVDs were displayed in Table 6. Among the 15,574 cited references, 10 documents were cited more than 50 times. The relationship of co-citation was established when two items were cited by another manuscript at the same time. Most of the co-cited references were articles and were related to the specific mechanism of ferroptosis involved in CVDs. The visualization of burst detection can also display the emerging trends and development of hot topics during the past years. Figure 6 vividly displayed the order of the strongest citation bursts from 2015 to 2022 by using CiteSpace. The extent of citation burst was measured by the frequency of citation and burst period. References with citation bursts first appeared in 2015, and the most recent references with citation bursts initiated in 2020. The strongest burst (strength: 8.88) appeared in 2015 for a 2019 (16).

**TABLE 6:** Top 20 co-cited articles.

Rank	Articles	Author year	Journal	IF	Type	Occurrences
1	Ferroptosis: an iron-dependent form of non-apoptotic cell death	Dixon et al. (18)	Cell	66.850	Article	170
2	Ferroptosis as a target for protection against cardiomyopathy	Fang et al. (29)	Proceedings of the national academy of sciences	12.779	Article	112
3	Regulation of ferroptotic cancer cell death by GPX4	Yang et al. (16)	Cell	66.850	Article	99
4	Ferroptosis: A regulated cell death nexus linking metabolism, redox biology, and disease	Stockwell et al. (82)	Cell	66.850	Review	87
5	Inactivation of the ferroptosis regulator Gpx4 triggers acute renal failure in mice	Friedmann Angeli et al. (23)	Nature cell biology	28.213	Article	86
6	Glutaminolysis and transferrin regulate ferroptosis	Gao et al. (41)	Molecular cell	19.328	Article	74
7	Ferroptosis: Process and function	Xie et al. (83)	Cell death And differentiation	12.067	Review	64
8	Protective effects of the mechanistic target of rapamycin against excess iron and ferroptosis in cardiomyocytes	Baba et al. (84)	American journal of physiology-heart and circulatory physiology	5.125	Article	60
9	ACSL4 dictates ferroptosis sensitivity by shaping cellular lipid composition	Doll et al. (85)	Nature chemical biology	16.174	Article	56
10	Oxidized arachidonic and adrenic PEs navigate cells to ferroptosis	Kagan et al. (86)	Nature chemical biology	16.174	Article	51
11	Ferroptosis as a p53-mediated activity during tumor suppression	Jiang et al. (87)	Nature		Article	45
12	Activation of the p62-Keap1-NRF2 pathway protects against ferroptosis in hepatocellular carcinoma cells	Sun et al. (88)	Hepatology		Article	42
13	RAS-RAF-MEK-dependent oxidative cell death involving voltage-dependent anion channels	Yagoda et al. (89)	Nature		Article	41
14	Puerarin protects against heart failure induced by pressure overload through mitigation of ferroptosis	Liu et al. (56)	Biochemical and biophysicaresearch communications		Article	41
15	The CoQ oxidoreductase FSP1 acts parallel to GPX4 to inhibit ferroptosis	Bersuker et al. (90)	Nature	69.504	Article	39
16	Synchronized renal tubular cell death involves ferroptosis	Linkermann et al. (91)	Proceedings of the national academy of sciences	12.797	Article	39
17	Ferroptosis: Death by lipid peroxidation	Yang and Stockwell, (92)	Trends in cell biology	21.167	Review	38
18	Synthetic lethal screening identifies compounds activating iron-dependent, non-apoptotic cell death in oncogenic-RAS-harboring cancer cells	Yang and Stockwell, (93)	Chemistry and biology	NA	Article	37
19	Autophagy promotes ferroptosis by degradation of ferritin	Hou et al. (94)	Autophagy	13.391	Article	37
20	Loss of cardiac ferritin H facilitates cardiomyopathy *via* Slc7a11-mediated ferroptosis	Fang et al. (95)	Circulation research	23.213	Article	36

### The analysis of burst keywords

**FIGURE 7:**
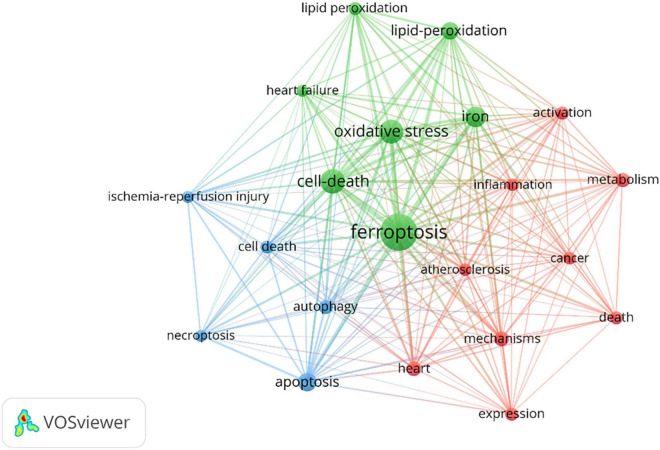
Visualization map of top 20 keywords.

The network construction of keywords makes it possible for us to quickly grasp research hotspots. Different keywords were classified into different clusters according to their categories and relationships. The more clusters, the more research directions. Table 7 displayed the high-frequency keywords. Most of the keywords were related to the specific processes that include oxidative stress, apoptosis, lipid-peroxidation, autophagy, and inflammation. Atherosclerosis, heart failure (HF), and ischemia-reperfusion (I/R) injury were the top three keywords related to the application of ferroptosis in CVDs. VOSviewer software were used to cluster these keywords. The circle and label represent an element, while the color indicates different clusters. Figure 7 displayed the clusters of red, blue, and green, indicating mainly three research directions. The green cluster mainly includes ferroptosis, cell death, oxidative stress, iron, heart failure, and lipid peroxidation. The blue cluster was ischemia-reperfusion injury, apoptosis, cell death, autophagy, and necroptosis. Red clusters were composed of atherosclerosis, inflammation, activation, and heart.

**TABLE 7:** Summary of the top 20 keywords related to ferroptosis in cardiovascular diseases.

Rank	Keyword	Occurrences	Total link strength	Rank	Keyword	Occurrences	Total link strength
1	ferroptosis	193	514	11	activation	25	101
2	cell-death	87	305	12	death	25	69
3	oxidative stress	180	2592	13	cell death	24	103
4	iron	60	251	14	expression	24	76
5	apoptosis	49	168	15	inflammation	23	85
6	lipid-peroxidation	43	170	16	cancer	23	80
7	heart	32	121	17	atherosclerosis	22	94
8	mechanisms	31	126	18	heart failure	21	81
9	autophagy	28	105	19	ischemia-reperfusion injury	20	88
10	lipid peroxidation	25	108	20	necroptosis	20	71

**FIGURE 8:**
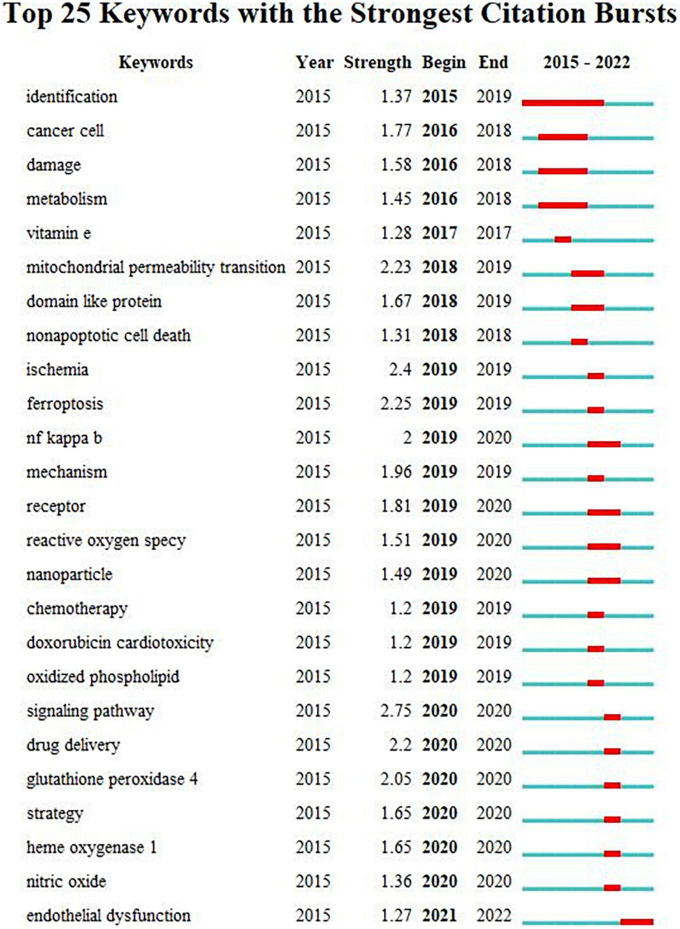
Top 25 keywords with the strong citation bursts.

**FIGURE 9:**
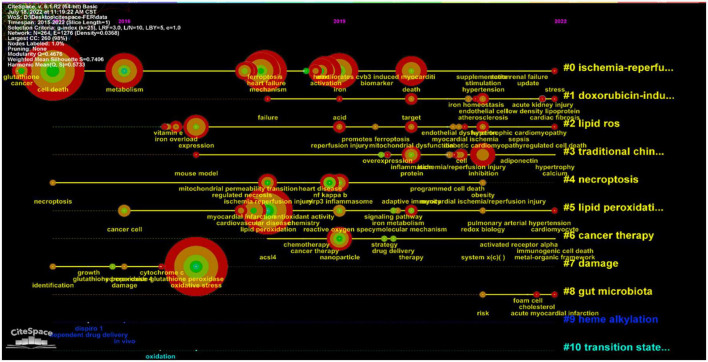
Timeline visualization map of the keywords.

Burst detection with strength and duration can reflect the emerging direction and trends. We also visualized the burst detection by VOSviewer (Figure 8). Over the past years, the signaling pathway ranked as the first with the maximum strength of 2.75, followed by ischemia (strength: 2.4), ferroptosis (strength: 2.25), mitochondrial permeability transition (strength: 2.23), drug delivery (strength: 2.2), glutathione peroxidase (strength: 2.05), and NF-kappa B (strength: 2). Moreover, endothelial dysfunction achieved the burst which had lasted from 2021 until now. We also constructed a timeline according to the distribution of keywords (Figure 9). I/R injury had always been the focus of ferroptosis in CVDs research. The study of ferroptosis in doxorubicin-mediated heart failure was also gradually entering public attention in 2018. Lipid peroxidation and the production of ROS was essential mediator during ferroptosis. At the same time, the relationship between gut microbiota, foam cells, cholesterol, and iron death gradually began to enter the public vision in recent years.

## Discussion

Cell death is an inevitable process during the cell cycle in all living beings. It has traditionally been believed that apoptosis and necrosis are the two main causes of cell death. Recently, an emerging trend of studies demonstrated that regulated cell death, such as necroptosis, proptosis, and ferroptosis, had gradually attracted public attention (17). Ferroptosis was first proposed by the author Dixon et al. in 2012. They discovered that a small molecule inhibitor can lead to specific non-apoptotic cell death, which can be inverted by iron chelators or lipophilic antioxidants (18). Although ferroptosis has only been proposed for 10 years, different forms of cell death relating to iron metabolism has already been widely debated in multiple research areas (19). The term of ferroptosis was defined as one of the regulated cell death processes in 2018 by the Nomenclature Committee on Cell Death which was different from other previous types of cell death. The characteristic was mainly related to glutathione peroxidase 4 (Gpx4) dysfunction in intracellular oxidative disorder (20). Recently, multi-investigation had proposed that various molecules and signaling pathways taking in the regulation of ferroptosis (21). Activated oxygen radicals and peroxides generated by iron’s redox properties accelerated cell senescence by destroying the stability DNA and disturb the repair systems which also known as ferritin aging (22). At the same time, the overaccumulation of iron can directly result in the production of ROS and lipid peroxidation which was triggered by the loss of lipid electrons on the cell membrane and over accumulation of free radical species such as peroxyl radicals, and hydroxyl radicals. Once the process of ferroptosis was triggered, cell will undergo a distinct necrotic morphology, characterized by a normal sized nucleus shrunk dysmorphic mitochondria with diminished crista, and intact or condensed membranes (23).

Ferroptosis has been debated in many literatures in recent years, showing an “exponential” rapid growth trend which was consistent with our study’s findings. Iron metabolism was crucial for normal cardiac function and disturbance of the iron balance was not uncommon in many CVDs (24). Iron participates in a variety of biological processes in cardiac function ranging from energy metabolism to nucleotide synthesis (25). It has been investigated that not only iron deficiency but also iron excess can cause direct myocardial damage.

Previous studies had demonstrated that iron deficiency was the most common malnutrition-associated disease and was found in more than 75% of patients with HF (26). Despite of the common results of anemia, iron deficiency can also result in fundamental metabolic change in cardiomyocyte. Lakhal-Littleton et al. reported that iron-deficiency associated anemia can lead to a metabolic remodeling toward a glycolytic, lactic acid-producing phenotype with a reduced flux through pyruvate dehydrogenase and elevated lactic acid production (27). Moreover, Ardehali et al. also discovered that the critical role of mRNA-binding protein tristetraprolin (TTP) in preventing the formation of impaired mitochondria in response to iron deficiency. They found that cardiac-specific depletion of TTP manifested normal phenotype at baseline condition but aggravated into spontaneous cardiomyopathy under iron deficiency and too much reactive oxygen species (ROS)-production in the mice hearts (28).

Excess iron deposition can lead to cardiac disorder by oxidative stress and ferroptosis *via* catalyzing the oxidation of phospholipid hydroperoxides on the cell membrane (29). Early studies determined oxidative stress was closely associated with CVDs and intervention that regulate these processes-particularly the antioxidant system of glutathione alleviated the incidence of cardiomyopathy (30, 31). The studies suggested that many CVDs were involved in ferroptosis. High levels of ferroptosis triggered by distinct signaling pathways can directly result in ischemic heart disease, HF and cardiomyopathy (32).

In addition to providing publications in specific areas, bibliographic sciences can accurately identify current hot topics and the most representative studies. Our study showed that authors from 36 countries had published relevant articles and experienced exponential growth. China held an absolute leading position in this field in terms of the number of literatures, institutions, or authors. Although the item of ferroptosis was first proposed by scholar from the USA, Chinese scientist’s explored ferroptosis and cardiovascular research most deeply. In the field of ferroptosis and CVDs, China, Germany, and the USA had made great contributions. From the authors distribution map, we can infer that the authors in related fields cooperated on a small scale and lacked large-scale close communications. J Peng, from Xiangya School of Pharmaceutical Sciences, published the most articles. The author is mainly engaged in the ferroptosis with I/R injury, and they had proposed that ferroptosis occurred mainly in the phase of myocardial reperfusion but not the ischemia period. Thus, the intervention of ferroptosis exerted beneficial effects on reperfusion injury which laid a solid foundation for precise therapy development in patients with myocardial infarction (33). The articles by Fang et al. and Min et al. achieved the highest citations. The two scholars exchanged and cooperated with each other and focused on the specific regulation of ferroptosis in the liver, heart, and brain. They also illustrated the molecular and metabolic landscape of iron and ferroptosis in CVDs and proposed that ferroptosis was a pivotal target for protection against cardiomyopathy (5, 29). S. J. Dixon was the first co-cited author who named the term of ferroptosis (18). The author also demonstrated the relationship between ferroptosis and cancer and brain injury (34, 35). The most co-cited reference was W. S. Yang, from Columbia University, whose manuscript achieved the most citations (16). The author identified Gpx4 was the center of ferroptosis which provided a potential target of ferroptosis-related compounds. M. J. Hangauer displayed that cancer cells were dependent on the lipid hydro peroxidase Gpx4 for survival and inactivation of Gpx4 results in selective ferroptosis *in vitro* and prevents tumor relapse in mice (36).

With the advance of technological development, bibliometric analysis can also reflect the dynamic fluctuation in specific fields. We can quickly understand and grasp the research progress and direction in this field by constructing keywords co-expression network. The top 10 keywords were mainly related to the specific regulation mechanism during the process of ferroptosis. I/R injury, HF, and atherosclerosis were the top three main directions in CVDs research.

### Ischemia/reperfusion injury

I/R injury is a fairly common, life-threatening complication in the cardiac disorder. The relationship of ferroptosis, and I/R has been reported in other organs such as the liver, brain and kidneys (22, 36, 37). Myocardial I/R injury refers to the pathological change of subsequent myocardial injury caused by reperfusion after acute coronary artery occlusion. Studies had demonstrated that cell death played a central role during I/R injury, particularly apoptosis, and necroptosis-associated cell death (38). Myocardial I/R injury was also closely related to ferroptosis. Zhang et al. had reported a ferroptosis inhibitor, polydopamine nanoparticles (PDA NPs) to alleviate cardio injury. The PDA NPs can limit the over production of Fe^2+^ and promote the activity of mitochondrial in H9c2 cells. They also discovered the treatment effect of PDA NPs by inhibiting Fe^2 +^ accumulation and lipid peroxidation in animal model (39). Tang et al. discovered that activation of transferrin receptor protein 1 (TfR1) by ubiquitin-specific protease 7 (USP7) and protein 53 (p53) triggered the incidence of ferroptosis and aggravated the I/R injury with elevated oxidized phosphatidylcholines (OxPCs), mitochondrial dysfunction, disturbed calcium transients, and variety cardiomyocyte death (40). Tang et al. discovered that the deceased expression of Gpx4 was proportion to the increased level of malondialdehyde (MDA) and iron concentrations during reperfusion in a rat model. They also proved ferroptosis occurred during the period of reperfusion instead of ischemia, which was consistent with the study of J Peng (33). Gao et al. discovered that eighter increased production of glutaminolysis or decrease level of iron can significantly alleviate I/R injury *via* repressing ferroptosis (41). Ma et al. discovered that overactivity of silent mating type information regulation 2 homolog- 1 (SIRT1) can alleviate I/R injury by inhibiting ferroptosis *via* p53/SLC7A11 signal pathway (42). Fang et al. found the protective role during both acute and chronic myocardial I/R injury by chelating iron or iron inhibitor Fer-1 indicting the potential therapeutic target during cardiac I/R injury (29). Another I-R injury during heart transplantation, which result in primary graft syndrome and increased mortality, was also associated with ferroptosis. Due to massive generation of endogenous cytokines by ferroptosis, a direct loss of cardiomyocytes taken place in the donor’s heart after neutrophils adhere to the coronary vessels (43). Not only iron in the lesion area promote the occurrence of ferroptosis, but residual myocardial iron was also a potential indicator of left ventricular remodeling both in human beings and animal study (8).

### Heart failure

Heart failure (HF) is the common end stage of various cardiac disorders. Death or loss of cardiomyocytes occupied a pivotal role in the progression of HF. The repaired tissue restored by fibrotic scar poses a great threat to people’s life. Many studies had illustrated that ferroptosis was associated with programmed cell death during HF (44). Iron accumulation in the heart will disturb vascular contraction or relaxation, aggravate the deterioration of atherosclerosis, and arrhythmias evenly HF (45). The most prominent hot topic related to ferroptosis is drug-induced HF- doxorubicin (DOX) induced cardiomyopathy (DIC). Since the late 1960s, anthracyclines such as doxorubicin, have been widely used in cancer treatment. However, the potent risk of cardiotoxicity had also restricted the widely applications.

Instead of the cardiomyocyte apoptosis, oxidative stress, and mitochondrial damage, recently studies also found that ferroptosis including iron overload was involved in DIC. Fang et al. proposed that ferrostatin-1 obviously alleviated DIC in animal study. They discovered that elevated level of heme oxygenase-1 (HO-1) in response to systemic DOX enhanced the generation of free iron and triggered ferroptosis (29). Moreover, the administration of Fer-1/deferasirox obviously increased the survival of mice exposed to DOX compared to other cell-death inhibitors. Another profound study made by Ardehali et al. had revealed that cardiotoxicity initiated through the preferential deposition of iron inside the mitochondria under doxorubicin treatment. Overexpression of ABC protein-B8 (ABCB8) both *in vitro* and *in vivo* study, a mitochondrial protein that promotes iron export alleviate mitochondrial iron accumulation and cellular ROS production (46).

Tadokoro et al. revealed that DOX restrained the expression of Gpx4 and promoted lipid peroxidation *via* Fe2 + complex, which also known as mitochondria-dependent ferroptosis (47). Acot1 (acyl-CoA thioesterase 1), mediated biosynthesis of polyunsaturated fatty acids (PUFAs) was closely associated with ferroptosis. Gene-depletion of acot1 made cardiomyocytes more sensitive to ferroptosis whereas overactivity of acot1 manifested notable protection effect (48). Zhang et al. found elevated levels of high-mobility group box 1 (HMGB1) by DOX, and over-expression of HMGB1 triggered the ferroptosis and cardiotoxicity which could also be offset by Fer-1 or Iron chelator dexrazoxane (DXZ) (49). Wang et al. reported that the protein level of arginine methyltransferase 4 (PRMT4) was down-regulated, while overexpression of PRMT4 promoted ferroptosis in DOX-treated cardiomyocytes, while the gene depletion or pharmaceutical inhibition displayed the converse results (50). Moreover, mice supplied with high iron or gene mutation, has higher risk of death after DOX administration, while an iron-deficient diet was accompanied by reduced incidence of DIC and elevated survival rate (51). The result implied that interference related to cardiac iron metabolism may serve as a potential method for drug-induced cardiomyopathy.

Another kind of HF related to ferroptosis was sepsis cardiomyopathy (SCM) which was common in patients with sepsis and systemic inflammatory response syndrome (52). It is believed that SCM is caused by myocardial depressant factors, including the release of bacterial toxins and cytokines production in the heart. Ferroptosis has been shown to be a contributing factor to the pathogenesis of SCM in previous studies. Li et al. found that increased levels of nuclear receptor coactivator 4 (NCOA4) produced by lipopolysaccharide (LPS) were proportional to increased level of Fe2 + and decreased level of ferritin. In addition, cytoplasmic Fe2 + promoted mitochondrial sideroflexin (SFXN1) production, leading to a greater amount of Fe2 + being over-accumulated in mitochondria, generating overproduction of ROS (53). Wang et al. reported that cecal ligation and puncture (CLP) increased myocardial injury accompanied by decreased Gpx4 and glutathione (GSH), and up regulated gasdermin D and iron concentrations (54). Cytokines such as interleukin (IL)-1β, tumor necrosis factor (TNF)-α, and HMGB1 can also directly promote ferroptosis and IL-1β had been proved to trigger ferroptosis in human umbilical vein endothelial cells (HUVECs) and diabetic endothelial cells (55). Moreover, ferroptosis had also been observed in pressure overload induced cardiac hypertrophy. Antioxidant Pueraria could directly restrain ferroptosis *via* Gpx4 and ferritin heavy chain 1 (FTH1). Moreover, the reduced level of nicotinamide adenine dinucleotide phosphate (NADPH) oxidase 4 (NOX4) can directly inhibit cell death both *in vivo* and in vitriol investigation (56). Although there have been many investigations elaborating the relationship between ferroptosis in HF triggered by ischemia, drug, inflammatory response, and mechanical stress, the specific regulation of ferroptosis in this type of cardiomyopathy remains to be fully explored.

Since ferroptosis was associated with lipid peroxidation, the dynamic change of lipid peroxidation is also involved in the development of obesity-related cardiomyopathy. Palmitic acid was found to inhibit the protein expression levels of GPX4 in a dose-and time-dependent manner and was restored by different ferroptosis inhibitors *in vitro* study (57). A recent study revealed that obese adipose tissue macrophages (ATMs) derived exosomes resulted in ferroptosis *via* glutathione synthesis disorder by targeting SLC7A11 in obesity-induced cardiac injury (58). Moreover, given the prominent role of mitophagy in the regulation of ferroptosis, Ren et al. found that FUN14 domain containing 1 (FUNDC1) deficiency sensitizes high fat diet-induced cardiac metabolic remodeling and abnormal contraction anomaly through ACSL4-mediated ferroptosis (59).

### Atherosclerosis and endothelial injury

Atherosclerosis (AS) belong to a chronic pathological progression founding on the large or middle arteries mediated by abnormal lipid accumulation, oxidative stress, and inflammation response. Ferroptosis has been involved during the whole process. Lipid accumulation, particularly poly unsaturated fatty acids (PUFAs), can directly result in oxidized lipids and inducing ferroptosis in the atherosclerosis plaque. The administration of Fer-1 to Apo E-/-mice on a high-fat diet (HFD) can promote solute carrier family 7 member 11 (SLC7A11) and Gpx4 expression, and partially inhibited lipid peroxidation and iron accumulation while increasing the viability of oxLDL-induced mouse aortic endothelial cells (MAECs) (51). Guo et al. demonstrated that overactivity of Gpx4 alleviate lipid peroxidation and progression in animal model (60). Bai et al. had demonstrated Fer-1 increased cell viability and reduced cell death in oxidized-low density lipoprotein (ox-LDL)-stimulated aortic endothelial cells (MAECs) in mice as well as ion chelator deferoxamine. Moreover, administration of Fer-1 down-regulated the activity of adhesion molecules and promote vasodilation function with elevated endothelial nitric oxide synthase (eNOS) activity. Unlike I/R injury, they proposed that ferroptosis might be involved during the whole progress of AS (51). Studies also demonstrated that the deposition of iron in normal arterial was lower than that in patients with atherosclerotic plaque (61). As iron deposition is a crucial factor during ferroptosis, preventing iron overload, particularly non-transferrin bound iron (NTBI), a deleterious form of iron, would be therapeutic target for atherosclerosis, too. One study reported that high systemic iron can result in endothelium disorder, which promoted the development of atherosclerosis in the end (45). Liu et al. found that selective induction of erythrocytosis with low-dose erythropoietin accelerated atherosclerosis with obvious ferroptosis, lipid peroxidation, and endothelial damage in VFEpoR mice. Liproxstatin-1, ferroptosis inhibitor l, inhibited increased atherosclerosis, lipid peroxidation, ferroptosis, and endothelial damage in VFEpoR mice (62).

Multi signal pathways related to ferroptosis have been debated in AS. The Kelch-like ECH-associated protein 1 (Keap1)-nuclear factor erythroid 2-related factor 2 (Nrf2)/antioxidant reaction element (ARE) pathway inhibited ferroptosis by keeping iron balance and accelerating glutathione, Gpx4, and NADPH production, while The Hippo pathway triggered the process of ferroptosis (63). Other critical genes mediate transcription activity, such as Activating transcription factors-3 (ATF3), ATF4, and signal transducer and activator of transcription 3 (STAT3), had also been implicated in ferroptosis and AS (64). As reported by Meng et al. in both her bioinformation and Elisa’s results, ferroptosis-related genes such as cyclin A2 (CCNA2), cyclin dependent kinase 1 (CDK1), and transferrin receptor 1 (TFRC) expression were obviously higher from coronary atherosclerosis samples in comparison with controls (65). Wu et al. revealed the ferroptosis-related gene heme oxygenase 1 (HMOX1) was highly expressed in atherosclerotic plaques accompanied by matrix metalloproteinases (MMPs) resulting in obvious M0 macrophages infiltration (66). Fernández-Garca reported that decreased concentration of iron in the spleen, liver, and heart in mice with splenic artery ligation. This decreased level of iron was proportion to an increased recruitment of F4/80^+^ -macrophages in the spleen which will recruit monocyte/macrophage in the atherosclerotic plaque (67).

### Endothelial dysfunction

The endothelial injury appears to be a new trend in atherosclerosis research, and it appears that it is closely related to ferroptosis. Researchers had reported erastin can result in ferroptosis in HUVECs (68). Endothelial cells may undergo cell death triggered by ferroptosis because of increased iron deposition and lipid overoxidation when the cell was exposed to PM2.5 (69). Cepharanthine inhibited 15-lipoxygenase-1 (ALOX15), which was proportional to a reduction in ferroptosis morphological changes in the mitochondria in the subarachnoid hemorrhage (SAH) model (70). According to Sheng et al. astragaloside IV prevented ferroptosis and promoted vascular regeneration by repressing the production of ROS in HUVECs (71). There were also study that iron deficiency accelerated the endothelial remodeling in the pulmonary vascular (72). However, the viewpoint that restricting iron ingestion may delay the hypoxia-induced endothelial remodeling in the lungs of mice had also been published (73). It had been widely accepted that inactivation of Gpx4 can directly triggered the ferroptosis, while gene depletion of Gpx4 was lethal both in embryonic and adult development (74, 75). In addition, conditional depletion of Gpx4 in endothelial cells cannot sustain a long time over 3 weeks exposed to thromboembolic events lacking vitamin E (76). These studies demonstrated the unsubstituted role of Gpx4 in CVDs. Although the specific role of Gpx4 with ferroptosis has not been interpreted clearly yet.

### Gut microbiota

Emerging evidence had illustrated the association between gut microbiota and CVDs *via* immune regulation, host energy metabolism, and oxidative stress. Studies depicting whether gut microbiota were involved in ferroptosis are relatively rare at present. Until recently, Robert et al. discovered that administration with omega-3 polyunsaturated fatty acids (n-3 PUFAs) and butyrate will promote mitochondrial Ca2 + -and Gpx4-dependent ferroptosis (77). Zhang et al. discovered that mercury exposure could induce neuronal ferroptosis and ferrostatin-1 can alleviate mercury-induced brain injury. While the perception that gut microbiota contribute to the pathological process of heavy metal-induced injury had always been reported (78). Deng et al. found that capsiate, the metabolite of gut microbiota, could promote Gpx4 expression and restrain ferroptosis *via* the overexpression of transient receptor potential vanilloid 1 (TRPV1) in intestinal I/R injury (79). A study by Tang et al. demonstrated that maternal embryonic leucine zipper kinase (MELK) inhibitor OTSSP167 significantly reduced inflammation of colitis, inhibited intestinal damage, and effectively retard the incidence colitis-driven carcinogenesis. At the same time. OTSSP167-mediated fecal microbiota transplantation effectively alleviated the severity of dextran sulfate salt (DSS)-induced colitis. Moreover, OTSSP167 can also inhibited the ferroptosis and suppressed macrophage infiltration and M1 polarization which is the key factor of the secreted pro-inflammatory factors (80). Based on the current research situation, the specific relationship of ferroptosis and gut microbiota in CVDs deserve further investigation.

### Limitations

Our study systematically summarized the relationship between ferroptosis and CVDs. However, some limitations existed in this study. First, all the literature was collected only from the WoSCC database which will result in negligence of studies from other databases. Second, non-English research literature was excluded, possibly resulting in source bias. The whole process was conducted by using bibliometric analysis based on machine learning, which may result bias as reported previously (81).

## Conclusion

Researchers found that cardiovascular research on ferroptosis is currently in a steady growth stage, and scientific research achievements are rising every year. At present, 36 countries around the world are engaged in this research. This field is dominated by China, followed by the United States, Germany, and Japan. The current research is generally scattered, and various countries lack stable and in-depth cooperation, which deserves our attention. In addition, Professor J Peng from China has made important contributions in this area. Professor W. S. Yang from the USA is the most cited scholar and achieved the strongest citation bursts. ferroptosis is most widely used in the fields of I/R injury, HF, and atherosclerosis. New attention has also been put on endothelial injury and gut microbiota, which could be the direction of future research. Further investigation upon potential ferroptosis inducers or inhibitors was a promising research topic for CVDs and other disorders related to ferroptosis.

## Data availability statement

The original contributions presented in this study are included in the article, further inquiries can be directed to the corresponding author.

## Author contributions

TT, C-YK, and Q-ZT designed the research. TT, C-YK, RH, and Z-GM collected the primary data. CH, XZ, and MH check and sort out the literature again. TT and C-YK analyzed the data and wrote the primary manuscript. Q-ZT wrote and revised the final manuscript. All authors listed have read and approved it for publication.
